# Paving the way for better ototoxicity assessments in cisplatin therapy using more reliable animal models

**DOI:** 10.3389/fncel.2025.1552051

**Published:** 2025-03-05

**Authors:** Vijayprakash Manickam, Marisa Zallocchi

**Affiliations:** Department of Biomedical Sciences, School of Medicine, Creighton University, Omaha, NE, United States

**Keywords:** clinical cisplatin, multi-cycle dosing, zebrafish ototoxicity, cisplatin behavioral testing, cisplatin optimization protocol

## Abstract

Cisplatin-induced hearing loss is a common and irreversible side effect affecting a significant proportion of cancer patients. While various strategies to mitigate this toxicity have been explored, there remains a critical need for effective treatments. A major challenge in developing new therapies is the lack of reliable animal models that accurately replicate the clinical use of cisplatin in humans, which typically involves multiple cycles of low-dose administration. Traditional models using high doses of cisplatin have resulted in high mortality and variable hearing loss, complicating the assessment of potential treatments. To address this, a multi-cycle model using lower cisplatin doses in mice was developed, providing hearing loss without mortality. However, variability in outcomes across different research groups persisted. In the present study, we optimize the multi-cycle model of cisplatin-induced ototoxicity by using clinical-grade cisplatin rather than laboratory-grade formulations. The use of clinical cisplatin ensures greater consistency, reliability, and relevance to human treatment protocols, as it adheres to the rigorous quality standards required for patient use. This new administration protocol will minimize variability across research laboratories and more accurately mimic the dosing regimens typically administered to cancer patients. Additionally, we have enhanced a zebrafish model for high-throughput screening of potential therapeutics, further improving the consistency of results. These improvements to the animal models are critical for accelerating the discovery and testing of therapies to prevent cisplatin-induced hearing loss, supporting the development of effective treatments for cancer patients undergoing cisplatin chemotherapy.

## 1 Introduction

Cisplatin-induced ototoxicity is an irreversible side effect that impacts up to 60% of patients with cancer. Since the introduction of cisplatin chemotherapy more than half a century ago (Ghosh, 2019), significant efforts have been directed toward eliminating this undesirable side effect (Breglio et al., 2017; Fernandez et al., 2019; Fernandez et al., 2021; Fung et al., 2018; Choeyprasert et al., 2013). Therapeutic strategies to prevent hearing loss include antioxidants targeting reactive oxygen species (ROS), lipid metabolism regulators, and thiol-based compounds that chemically deactivate cisplatin (Fernandez et al., 2021; Kros and Steyger, 2019; U.S. Food and Drug Administration, 2022). Despite these efforts, the U.S. Food and Drug Administration (FDA) has only recently approved sodium thiosulfate (STS) as the first therapy to protect against cisplatin-induced hearing loss (CIHL) in a limited number of pediatric cancer patients (U.S. Food and Drug Administration, 2022). Therefore, there remains an unmet need for alternative therapies that can reduce CIHL while enabling patients to successfully complete their cancer treatment.

A significant challenge in developing novel therapies for CIHL has been the lack of an animal model that consistently recapitulates cisplatin treatment in humans. In earlier studies, researchers used higher single doses of cisplatin (>30 mg/kg b.w.) to induce ototoxicity in mice (Hazlitt et al., 2018; Ramkumar et al., 2021). However, this approach resulted in high mortality rates and, more importantly, significant variability in the animals’ hearing sensitivity, making it difficult to draw reliable conclusions about therapeutic interventions. Additionally, the single-dose treatment did not reflect the multiple cycles of lower-dose cisplatin typically used in cancer patients (Fernandez et al., 2019).

Recently, Fernandez et al. published an optimized protocol for cisplatin ototoxicity that more closely mimics the treatment given to cancer patients. In this multi-cycle protocol, 8-weeks-old CBA/CaJ mice received low doses of cisplatin (2.5 mg/kg to 3.5 mg/kg) for four consecutive days (Fernandez et al., 2019; Breglio et al., 2017), followed by 10 days of recovery in a three-cycle regimen. Using this protocol, the authors achieved consistent hearing threshold elevations with zero mortality. Furthermore, this animal model provided a clinically relevant system for studying potential therapies aimed at preventing hearing loss in cancer patients.

In this study, we further optimized the multi-cycle protocol. Fernandez’s method involved in-house preparation of cisplatin for daily administration, requiring weighting the cisplatin powder and dissolving it in saline solution at 37°C. This process introduced variability in the procedure across different research groups. We addressed this issue by standardizing the cisplatin working solution with the clinical formulation used for cancer patients. This cisplatin is subject to the rigorous quality and purification standards as set forth by the FDA that is required for clinical use in humans. In our optimized protocol, we provide guidance on animal husbandry during treatment.

Additionally, we have also optimized a zebrafish model for cisplatin-induced ototoxicity using clinical cisplatin. This model enables high-throughput screening of potential therapeutic compounds with enhanced consistency across laboratories.

We anticipate that these modifications to the previous protocol will improve outcomes and consistency among research groups, thereby accelerating the discovery of novel therapies for the protection and prevention of cisplatin-induced ototoxicity.

## 2 Materials and methods

### 2.1 Study design

The objective of this study was to optimize a model for clinical cisplatin-induced ototoxicity. In the case of the mouse studies, after the first round of hearing recordings (baselines) and confirmation of similar hearing sensitivity, animals were randomly assigned to the different groups with an equal number of females and males (6–8 mice per group). Similarly, 5–6 days post-fertilization (dpf) zebrafish were randomly allocated to different treatments (6 fish per group). Sample sizes were estimated based on previous work (Zallocchi et al., 2024; Vijayakumar et al., 2024; Ingersoll et al., 2020). Animals were examined daily and humanely euthanized at defined study endpoints. Each animal (mouse or fish) was considered as an independent biological replicate.

### 2.2 Animals

Experiments in mice (CBA/CaJ) and zebrafish (TuAB) were performed according to Creighton University’s standard methods, approved by the Institutional Animal Care and Use Committee (IACUC protocols #1241 and #1097).

Mice were housed under a 12-h light/12-h dark cycle, with food and water *ad libitum* in Creighton University’s Animal Resource Facility. Fish were maintained at 28.5°C in E3 media (5 mM NaCl, 0.17 mM KCl, 0.33 mM CaCl_2_, and 0.33 nM MgSO_4_, pH 7.2) (Westerfield, 2022).

### 2.3 Clinical cisplatin treatment

#### 2.3.1 Mice

The clinical cisplatin (Accord, NDC 16729-288-11, Creighton’s Pharmacy) ototoxicity model is an optimization of the previously described by Fernandez et al. (2019), with the following modifications: Experimental mice were provided with dry chow, food supplements and hydration gel upon weaning. We initiated treatment when animals were 7–8 weeks old and weighed at least 20 grams. The experimental groups were as follows: Age-matched control (no treatment), vehicle (saline solution), and clinical cisplatin (2 mg/kg b.w., 2.5 mg/kg b.w. or 3 mg/kg b.w.). Clinical cisplatin was administered once a day (between 9:00 AM to 10:00 AM) for four consecutive days in a three-cycle treatment, with 10 days of recovery between cycles. Animals received 0.5 mL of subcutaneous saline solution twice daily during the procedure and for 1 week post-treatment. Body weight was periodically recorded and animals were sacrificed 60 days after initiation of the treatment with their inner ears microdissected and processed for confocal analysis.

#### 2.3.2 Zebrafish

Five- to six dpf zebrafish were incubated with clinical cisplatin (100 μM to 600 μM) or vehicle (saline solution, control) in E3 media for 6 h. After 30 min recovery in regular E3 media, the fish were sacrificed and processed for immunohistochemistry (Zallocchi et al., 2021).

### 2.4 Hearing tests

Auditory brainstem response (ABR) recordings and data management were performed using a Tucker-Davis Technologies (TDT, Alachua, FL) RZ6 Multi I/O processor and BioSigRZ software (Zallocchi et al., 2024). The TDT MF1 transducer presenting the stimuli, was placed 10 cm from the left ear of the animals. Tone bursts of 5 msec duration at half-octave frequency intervals from 4.0 kHz - 45 kHz were presented at a rate 19/s. The sound intensity ranged from 20 to 100 dB SPL in 5 dB steps.

For DPgrams and distortion product otoacoustic emissions (DPOAEs) the primary tones f1 and f2 were generated using the TDT hardware. Recordings were made as level/frequency functions, with f2/f1 fixed at 1.2, and the level of the f2 (L2) 10 dB lower than f1 (L1). The signal was collected by a DP600 microphone (TDT systems) (Zallocchi et al., 2024).

### 2.5 Behavioral paradigm and stimulus response

Our experimental paradigm was adapted from Han et al. (2020), Beppi et al. (2021), and Basnet et al. (2019), consisting of four vibratory stimulations of 100 msec each, with a 60-s recovery period between stimuli. The stimulus frequencies were 90 Hz, 190 Hz, 310 Hz and 400 Hz, that are within the range of described oscillating water frequencies detected by superficial and canal neuromasts (Coombs et al., 2014).

Zebrafish were exposed to vehicle (saline solution) or 400 μM of clinical cisplatin, and their behavioral response was assessed 24 h after treatment. Individual 5–6 dpf zebrafish larvae were placed in 12-well plates containing 2 mL of E3 media and acclimatized before the initiation of the recordings for at least 30 min. The response to stimuli was tested by introducing vibration pulses at increasing frequencies using a ZebraBox system (ViewPoint) (Young et al., 2022; Beppi et al., 2021). Environmental light levels (~300 Lx) were maintained during the tests, and water temperature was kept at 28.5°C. The cumulative distances traveled (CDT) over the successive stimuli and during the ~4 min testing period were calculated for each fish (Han et al., 2020; Beppi et al., 2021; Basnet et al., 2019).

### 2.6 Immunohistochemistry

#### 2.6.1 Mice

The inner ears were microdissected and fixed in 4% paraformaldehyde (PFA) in Sørensen’s buffer (0.1 M pH 7.4) for 20 min at 4°C. After three washes in PBS and decalcification in EDTA (150 mM), the organ of Corti was isolated and immunostained as previously described (Zallocchi et al., 2024; Manickam et al., 2023). Briefly, the samples were blocked for 1 h at room temperature in PBS with 0.1% Triton X-100 (PBSTx) containing 10% of normal horse serum, and then incubated overnight at 4°C with the primary antibodies in blocking solution. After several washes in PBSTx, the samples were incubated with Alexa-Fluor conjugated antibodies at 1:500 for 2 h at room temperature, mounted using Prolong Gold mounting media (Thermo Fisher), and imaged.

#### 2.6.2 Zebrafish

5–6 dpf fish were processed as previously described (Zallocchi et al., 2024). Briefly, animals were fixed in 4% PFA overnight at 4°C with rocking. After washing with PBSTx, the samples were incubated with mouse anti-otoferlin (1:200) in blocking solution (PBSTx +3% BSA + 3% FBS) overnight at 4°C. The following day, fish were washed, incubated with Alexa-Fluor conjugated antibodies (1,500 dilution), and mounted using Prolong Gold mounting media (Thermo Fisher).

### 2.7 Antibodies

Rabbit anti-myosin 7A (Proteus Biosciences, 25–6,790), 1:200 dilution; mouse anti-CtBP2 (BD Biosciences, 612,044), 1:200 dilution; and mouse anti-hair cell soma (otoferlin, Developmental Studies Hybridoma Bank, HCS1), 1:200 dilution.

### 2.8 Imaging

Confocal imaging was performed using a Zeiss LSM 980 confocal laser scanning system with a 63x oil immersion objective (Carl Zeiss). Images were captured at room temperature with automatic sectioning, and data were processed using ZEN Blue edition software. *Z*-stack images are presented as flat *Z*-projections. Only linear adjustments to brightness and contrast were made, and final figures were assembled using Adobe Photoshop and Illustrator software.

### 2.9 Data acquisition and statistical analysis

#### 2.9.1 Mouse studies

Outer hair cells (OHCs) were manually counted, while CtBP2 puncta was quantified using Imaris (version 9.0). Frequencies along the organ of Corti were mapped using the Measure Line plugin from ImageJ (NIH), and OHC and ribbon synapse quantification was performed for the 16 kHz, 32 kHz and 45 kHz regions. Results were expressed as the number of OHCs per 100 μm of organ of Corti and number of ribbon synapses per inner hair cell (IHC). Each animal (mouse or fish) was an independent biological replicate.

#### 2.9.2 Zebrafish studies

For hair cell quantification, at least three neuromasts were inspected in a total of 6 zebrafish per treatment group. The average number of hair cells per neuromast was plotted for each animal. The neuromasts inspected were part of the cranial system and included the otic, middle, and opercular neuromasts. Hair cells were manually counted using a Zeiss Axiovert A1 Research Inverted Microscope with a 40X objective. In the figures, results are presented as the average number of hair cells per neuromast per fish (total of six fish). For the behavioral test, eight fish were used per treatment group. Results were expressed as the CDT during the stimulus period or the total experimental time (~4 min). Each animal was considered an independent biological replicate.

#### 2.9.3 Statistical analysis

ABRs, DPgrams and DPOAEs were analyzed by two-way ANOVA followed by Dunnett’s multiple comparison test. One-way ANOVA or Student’s *t*-tests were used for the remaining experiments. Results are presented as mean +/− SD with individual data points. The number of independent biological replicates is indicated in the figures or figure legends. All statistical tests were performed using GraphPad Prism (version 9.3.1). *p* values less than 0.05 were considered significant. Final figures were assembled using Adobe Photoshop.

## 3 Results

### 3.1 Assessment of clinical cisplatin ototoxicity in mice

**Figure 1:**
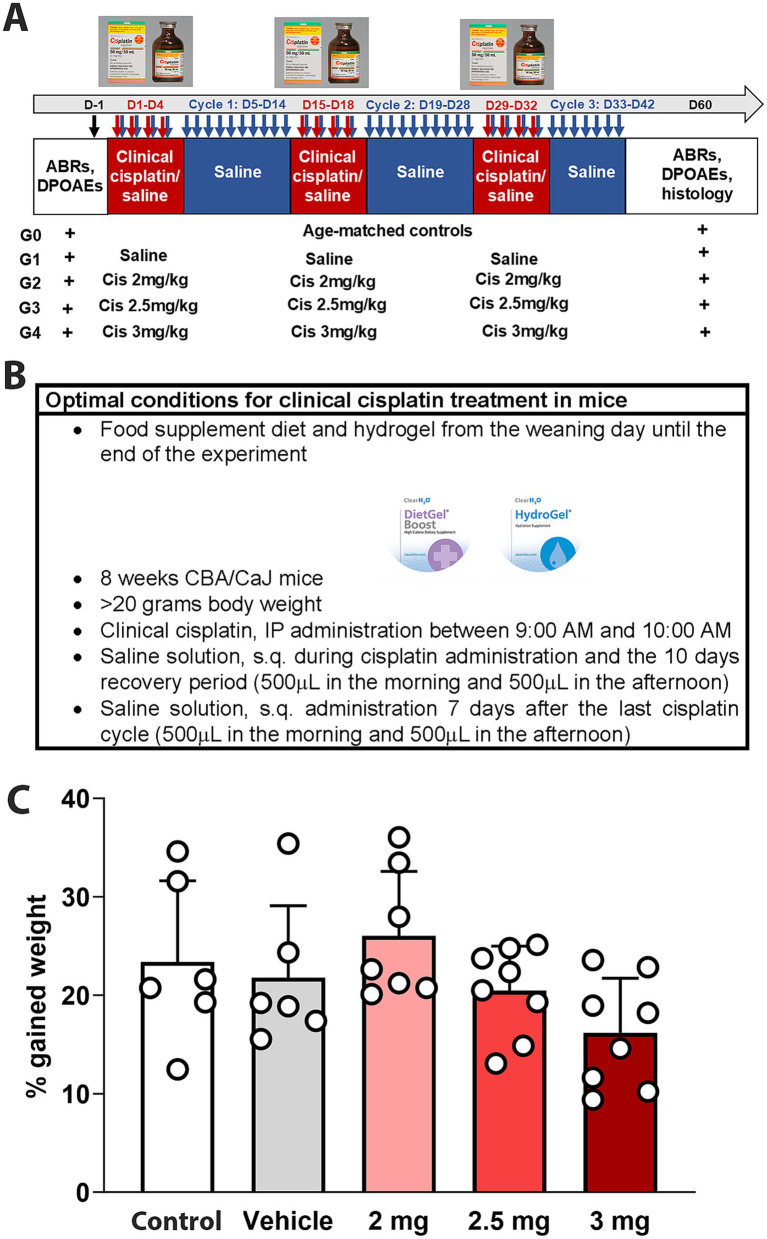
Experimental design for multi-cycle clinical cisplatin protocol. **(A)** Administration protocol for clinical cisplatin including the experimental groups. **(B)** Optimal condition for clinical cisplatin treatment. **(C)** Percentage of gain weight 60 days after the initiation of clinical cisplatin treatment. There were no significant differences between the treatments. Each circle represents an animal. Results are expressed as Mean +/− SD.

CBA/CaJ mice were treated with different doses of clinical cisplatin (2 mg/kg b.w., 2.5 mg/kg b.w., or 3 mg/kg b.w.) during a 3-cycle treatment regimen (Figures 1A,B). Hearing tests were conducted before and 60 days after the initiation of cisplatin treatment, and body weights were recorded daily throughout the treatment. None of the animals in any of the treatment groups showed significant weight loss by the end of the protocol, indicating that clinical cisplatin did not cause general toxicity (Figure 1C). Although the percentage of weight gained was reduced in the group receiving the highest dose of cisplatin, this difference was not statistically significant when compared to vehicle or age-matched controls. Regarding animal behavior, a reduction in physical activity and grooming was observed during the third cisplatin cycle, but only in the 2.5 mg/kg and 3 mg/kg dose groups. These abnormal behaviors were rapidly recovered within the following 10 days post-cisplatin treatment.

**Figure 2:**
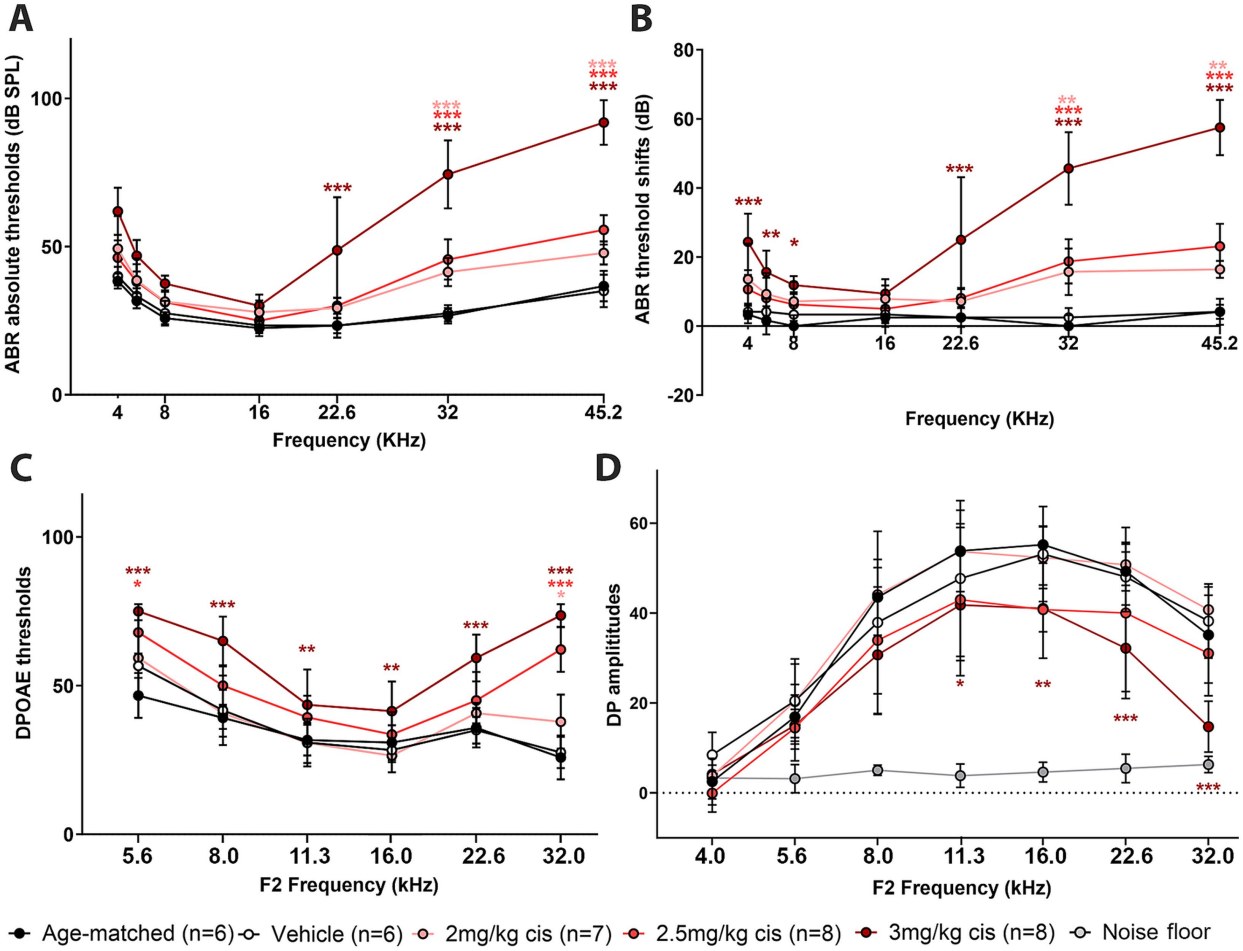
Clinical cisplatin affects hearing function in mice in a dose-dependent manner. Eight weeks old mice (>20 grams) received clinical cisplatin in a multi-cycle protocol. Hearing function was measured before and after the completion of cisplatin treatment. The following groups were tested: age-matched controls (black circles), vehicle (saline solution, white circles), clinical cisplatin at 2 mg/kg b.w. (pink circles), 2.5 mg/kg b.w (red circles) and 3 mg/kg b.w. (marron circles). **(A)** ABR thresholds. **(B)** ABR threshold shifts. **(C)** DPOAEs thresholds. **(D)** DP amplitudes. Noise floor gray circles. Results are expressed as Mean +/– SD. Statistical analysis: Two-way ANOVA followed by Dunnett post-test for multiple comparisons. *p < 0.05, **p < 0.01, and ***p < 0.001 vehicle *versus* the corresponding color-coded treatment. Number of animals per group is between brackets.

ABRs, DPOAEs and distortion product (DP) amplitudes were affected in a dose-dependent manner (Figures 2A–D). In the case of ABRs and ABR threshold shifts, all three cisplatin doses caused a significant increase at higher frequencies (32 kHz and 45 kHz), while only the 3 mg/kg b.w. dose resulted in significant elevations at intermediate and low frequencies. DPOAEs, a measurement of OHC function integrity, were also increased by clinical cisplatin, with the 3 mg/kg b.w. dose causing significant elevations at all the tested frequencies (Figure 2C). Conversely, DPOAE amplitudes were reduced by clinical cisplatin treatment (Figure 2D).

Overall, hearing tests confirm the dose–response deleterious effect of clinical cisplatin, with higher doses causing severe impairment.

**Figure 3:**
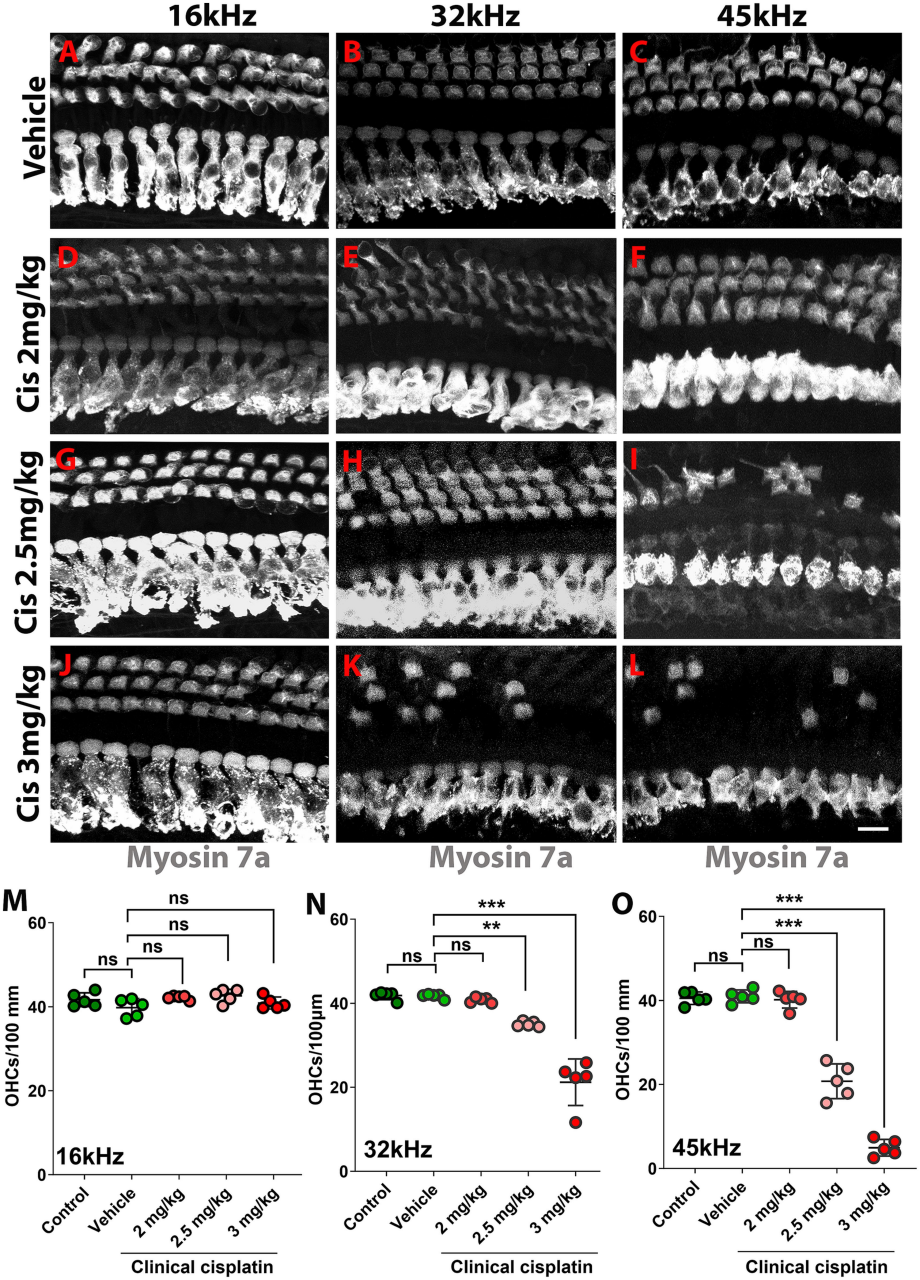
Clinical cisplatin treatment results in hair cell damage. **(A–L)** Representative micrographs of the organ of Corti at three frequency regions (16 kHz, 32 kHz and 45 kHz). Hair cells were immunostained for myosin-7a (gray). **(A–C)** Vehicle. **(D–F)** Clinical cisplatin 2 mg/kg b.w. **(G–I)** 2.5 mg/kg b.w. **(J–L)** 3 mg/kg b.w. Scale bar = 10 μm. **(M–O)** OHC quantification at 16 kHz **(M)**, 32 kHz **(N)** and 45 kHz **(O)** regions. Mean +/− SD. Circles represent individual animals. Statistical analysis: One-way ANOVA followed by Dunnett post-test for multiple comparisons. **p < 0.01, ***p < 0.001 *versus* vehicle. ns, not significant.

The hearing test results were complemented by morphological analysis (Figure 3). The organs of Corti from the different treatment groups were isolated 60 days post-treatment and immunostained for the hair cell marker myosin 7A (Figures 3A–L). At the lowest cisplatin dose (2 mg/kg b.w.), no hair cell loss was observed at any frequency region (Figure 3M). However, the number of OHCs was significantly reduced with the 2.5 mg/kg b.w. and 3 mg/kg b.w. cisplatin doses. Inspection of the 32 kHz and 45 kHz regions showed significant loss of OHCs compared to vehicle and age-matched controls (Figures 3N,O). The 16 kHz region was used as a control, as ABR thresholds were preserved and no OHC loss was observed.

**Figure 4:**
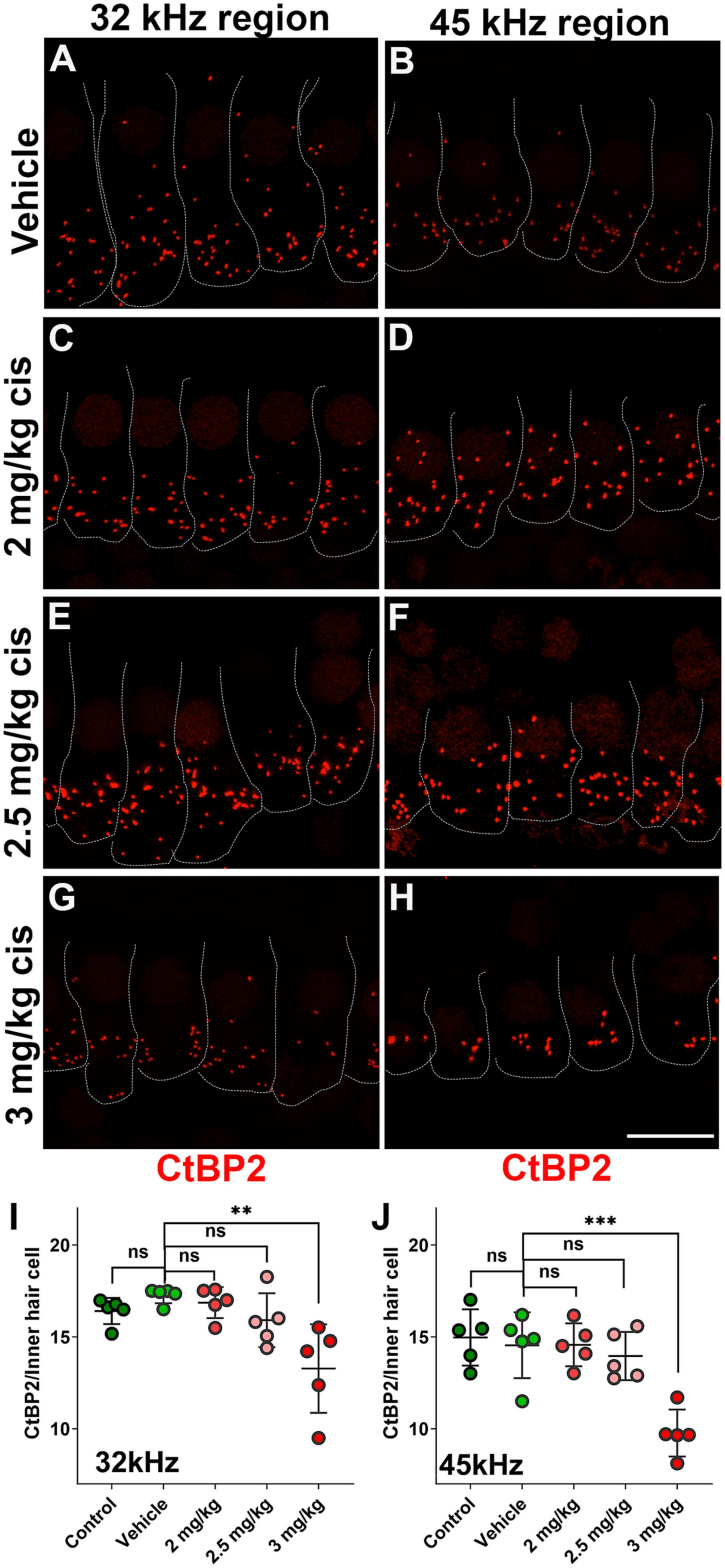
Clinical cisplatin treatment results in pre-synaptic ribbon loss at higher frequencies. **(A–H)** Representative micrographs of the organ of Corti at two frequency regions (32 kHz and 45 kHz). Pre-synaptic ribbons were immunostained for CtBP2 (red). Hair cells were delineated with white traces. **(A,B)** Vehicle. **(C,D)** Clinical cisplatin 2 mg/kg b.w. **(E,F)** 2.5 mg/kg b.w. **(G,H)** 3 mg/kg b.w. Scale bar = 10 μm. **(I,J)** pre-synaptic ribbon quantification at 32 kHz **(I)** and 45 kHz **(J)** regions. Mean +/− SD. Circles represent individual animals. Statistical analysis: One-way ANOVA followed by Dunnett post-test for multiple comparisons. **p < 0.01, ***p < 0.001 *versus* vehicle. ns, not significant.

The pre-synaptic buttons were also inspected by CtBP2 immunostaining (Figure 4). There was a significant reduction in the number of ribbons at higher frequencies (32 kHz and 45 kHz) following treatment with 3 mg/kg b.w. of clinical cisplatin (Figures 4G–J). In contrast, no significant reduction in the number of ribbons was detected at the lower cisplatin doses when compared to vehicle-treated animals (Figure 4A–F,I,J).

**Figure 5:**
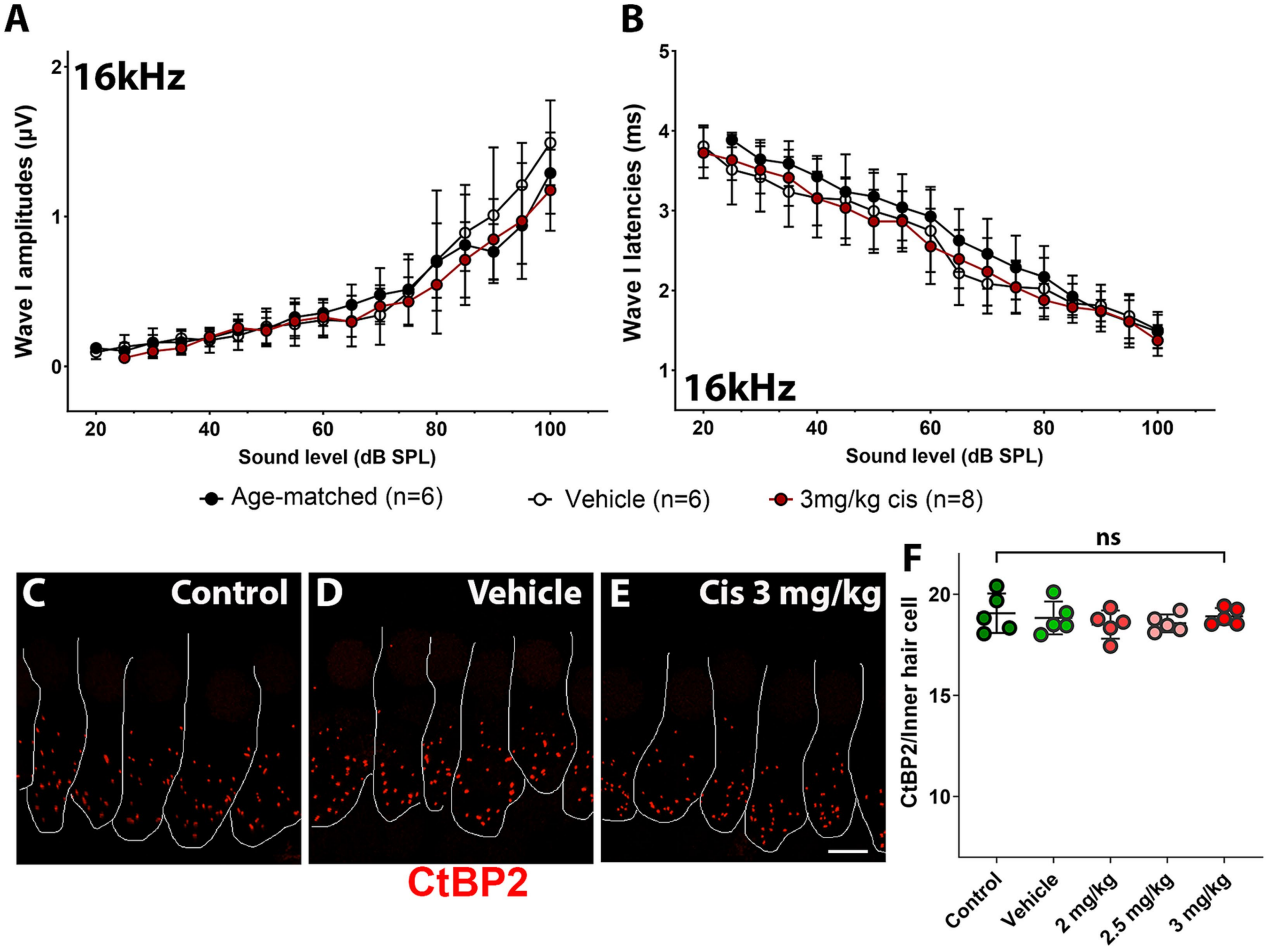
Assessment of clinical cisplatin treatment at the 16 kHz frequency region. Wave I amplitudes **(A)** and latencies **(B)** were analyzed for the 16 kHz region in age-matched control animals (black), and animals receiving saline solution (vehicle-control, white) or clinical cisplatin (3 mg/kg b.w., maroon). **(C–E)** Representative micrographs of the organ of Corti at the 16 kHz frequency region. Pre-synaptic ribbons were immunostained for CtBP2 (red). Hair cells were delineated with white traces. **(C)** Age-matched control. **(D)** Vehicle control. **(E)** Clinical cisplatin 3 mg/kg b.w. Scale bar = 5 μm. **(F)** Pre-synaptic ribbon quantification at the 16 kHz region for all the groups. Mean +/− SD. Circles represent individual animals. Statistical analysis: One-way ANOVA followed by Dunnett post-test for multiple comparisons. ns, not significant.

At the 16 kHz frequency, while ABR recordings (Figures 2A,B) and OHC numbers (Figure 3M) remained unchanged even at the highest cisplatin dose, significant alterations were observed in DPOAE thresholds (Figure 2C) and amplitudes (Figure 2D) relative to the vehicle-treated group. As expected, analysis of the wave I amplitudes and latencies (Figures 5A,B) at this frequency, along with CtBP2 quantification (Figures 5C–F), confirmed the absence of detectable defects at the IHC level, both morphologically and functionally. These results suggest that although OHCs (and pre-synaptic ribbons) remain present in the 16 kHz frequency region, they are functionally impaired, which accounts for the elevated DPOAE thresholds and reduced amplitudes (Figures 2C,D). It is plausible that with enough time, OHCs in the 16 kHz region may undergo degeneration.

Overall, these results demonstrated that, at the tested doses, clinical cisplatin treatment did not cause general toxicity but impaired hearing function in a dose-dependent manner. The 3 mg/kg b.w. dose resulted in a 40–60 dB threshold shift at the higher frequencies (32 kHz to 45 kHz), with the hearing deficits strongly correlated with the loss of OHCs and pre-synaptic ribbons.

### 3.2 Assessment of clinical cisplatin ototoxicity in zebrafish

Zebrafish are a useful model for studying ototoxicity because they can detect vibrations via the lateral line system, which is composed of hair cells arranged in rosette-like structures called neuromasts (Owens et al., 2007). Neuromasts are located on the surface of the fish, facilitating drug exposure, and making zebrafish an ideal model for high-throughput screening of otoprotective compounds (Vlasits et al., 2012; Owens et al., 2007; Zallocchi et al., 2021). Cisplatin-induced ototoxicity has been well-documented in zebrafish larvae, with many research groups currently facing similar challenges as in the mouse model, including variability in cisplatin preparation and solubility (Zallocchi et al., 2021; Lee et al., 2022a). To address these issues, we optimized a zebrafish model for cisplatin ototoxicity using clinical cisplatin.

**Figure 6:**
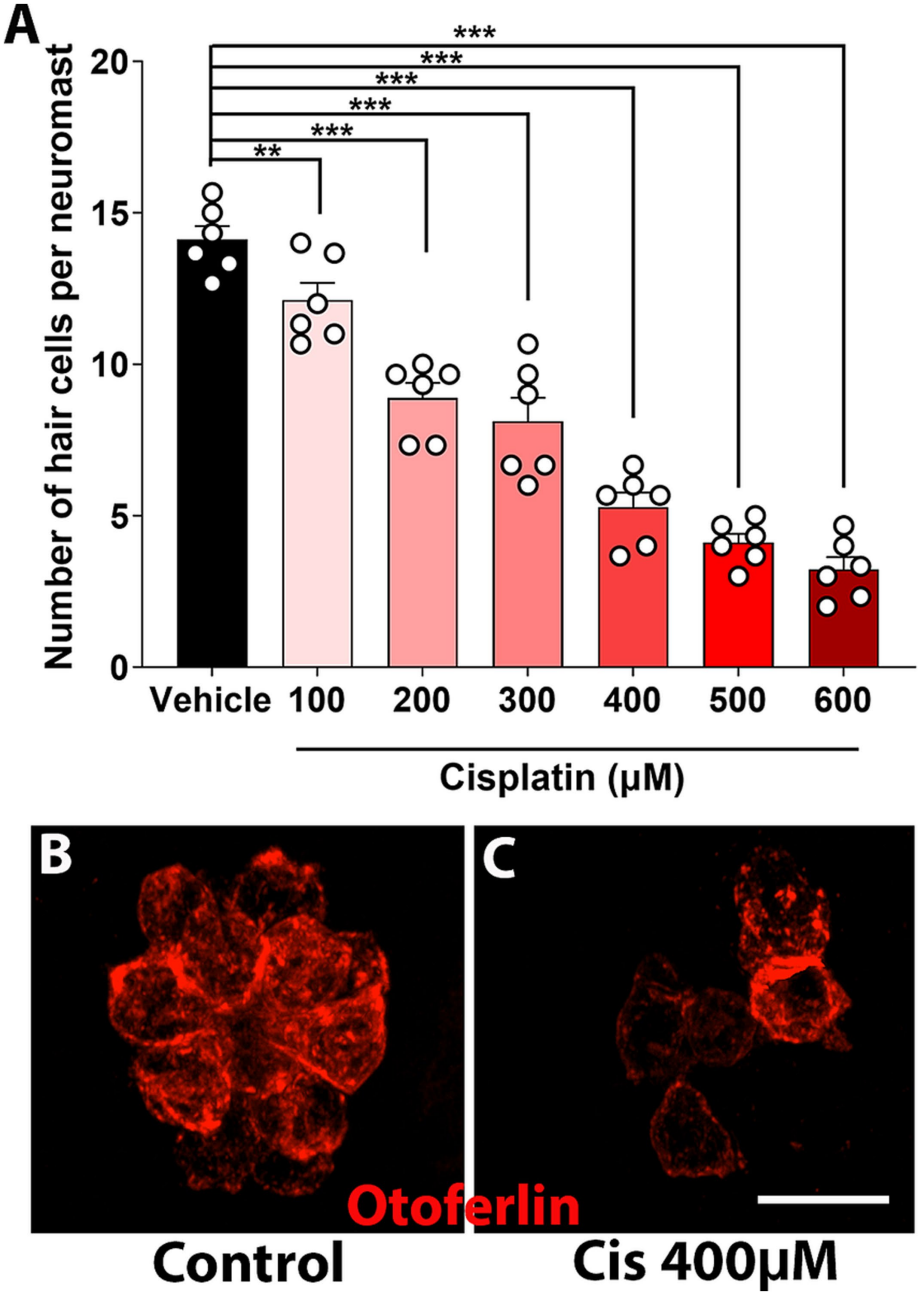
Dose–response curve of clinical cisplatin in zebrafish. **(A)** Quantitative data obtained from 5 to 6 dpf zebrafish treated with vehicle (saline solution) or increasing concentrations of clinical cisplatin (100 μM to 600 μM) for 6 h. Results are expressed as number of hair cells per neuromast. Mean +/− SD. Circles represent individual animals. Statistical analysis: One-way ANOVA followed by Dunnett post-test for multiple comparisons. **p < 0.01, ***p < 0.001 *versus* vehicle. **(B,C)** Representative images of a neuromast from a fish treated with vehicle **(B)** or clinical cisplatin at the EC_50_ (**C**, 400 μM). Hair cells were immunostained for otoferlin (red). Scale bar = 10 μm.

Five-to-six days dpf zebrafish were incubated with increasing concentrations of clinical cisplatin (100 μM to 600 μM) for 6 h, and the number of neuromast hair cells was assessed (Figures 6A–C). A dose–response effect was observed (Figure 6A), with an EC_50_ of 300 μM – 400 μM for clinical cisplatin. Notably, zebrafish incubated with 600 μM cisplatin, although still alive at the end of the incubation, exhibited a reduction in the swimming behavior, likely due to the significant loss of neuromast hair cells.

**Figure 7:**
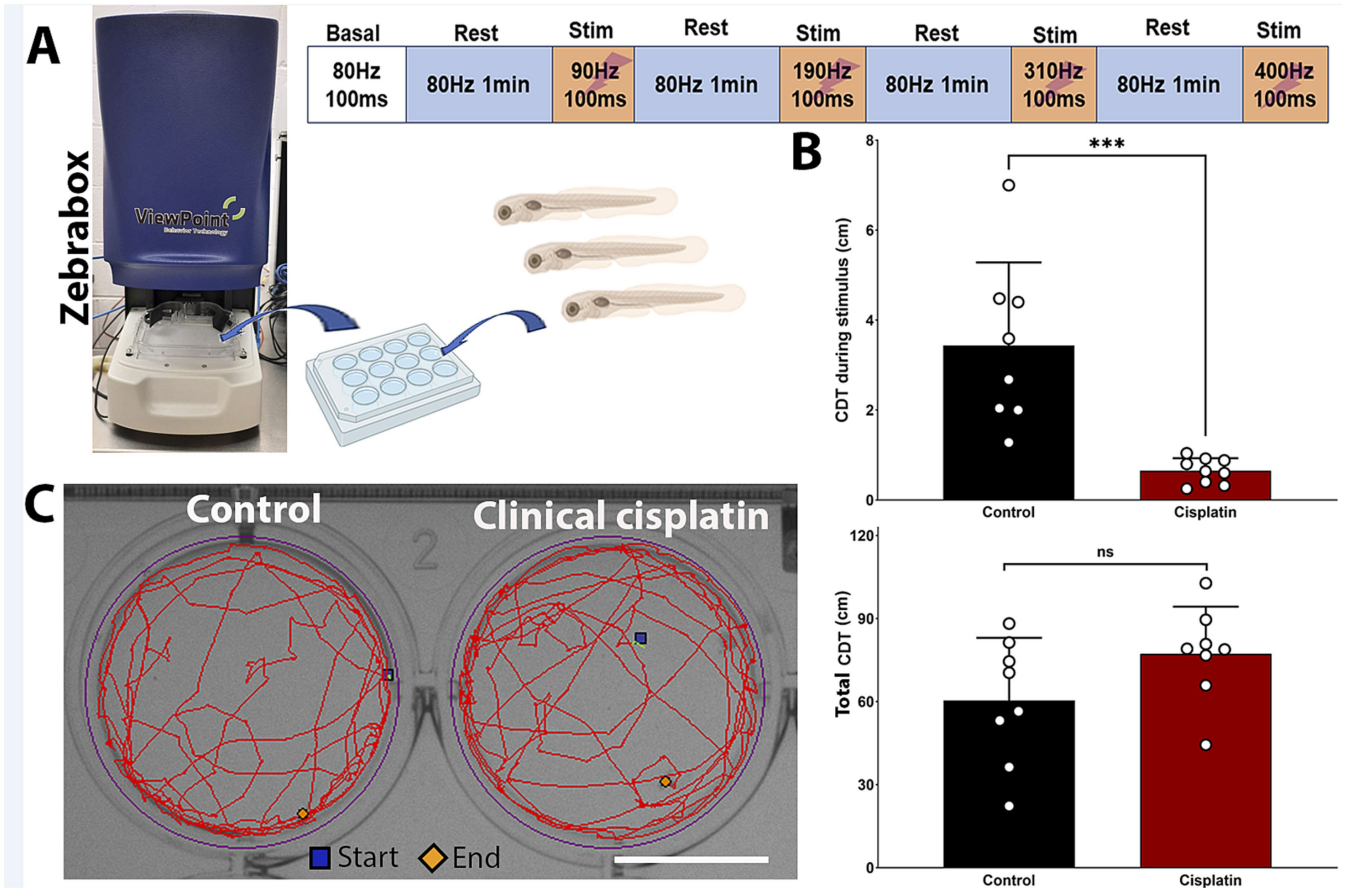
Clinical cisplatin treatment affects stimulus-dependent swimming behavior. **(A)** Experimental setup for the behavioral studies. **(B)** Cumulative distance traveled (CDT) by the zebrafish larva during the four stimulus periods (top) or over the 4 min experimental period (bottom). Results are expressed as mean +/− SD. Circles represent individual animals. Statistical analysis: Student’s *t* test. ***p < 0.001. **(C)** Track visualization of the distance covered over the 4 min period by larva treated with vehicle or clinical cisplatin. Scale bar = 1.25 cm. Biorender was used to generate part of this figure.

The lateral line system plays a key role in swimming behaviors such as rheotaxis, prey detection, and predator avoidance, making zebrafish behavior a secondary indicator of ototoxicity (Coombs et al., 2014; Mo and Nicolson, 2011; Seiler et al., 2005). The ZebraBox automated imaging system (ViewPoint, Figure 7A) can track zebrafish movement post-treatment, serving as a behavioral readout for neuromast damage.

Zebrafish larvae were exposed to 400 μM clinical cisplatin for 6 h, with swimming behavior evaluated 24 h later (Figure 7). The larvae were exposed to increasing stimulus frequencies (90 Hz to 400 Hz) for 100 msec, with a 1-min recovery period (Figure 7A). This behavioral protocol was adapted from previous studies with a few modifications (see Materials and Methods) (Han et al., 2020; Beppi et al., 2021; Basnet et al., 2019). We used the calculated CDT during the four stimulation periods to assess whether there was significant differences between control and cisplatin-treated fish (Figure 7B). Larvae treated with clinical cisplatin showed a significant reduction in their response compared to controls. This was evident by the decrease in the CDT during the stimulus (Mean +/– SD: control (*N* = 8): 3.434 +/− 1.851 cm *versus* clinical cisplatin (*N* = 9): 0.650 +/− 0.281 cm) (Figure 7B). These results suggest that after clinical cisplatin treatment, the intensity of the response elicited by the short frequency stimuli is decreased, likely due to damage to the hair cells.

Furthermore, there were no significant differences in the total CDT (~4 min) between the control and clinical cisplatin-treated groups (Figures 7C,D), indicating that the primary effect of clinical cisplatin was on the hair cells rather than the fish muscular activity (Mean +/– SD: control (N = 8): 60.346 +/− 22.700 cm *versus* clinical cisplatin (N = 9): 77.245 +/− 17.033 cm). Notably, the distances obtained for the total CDT (Figure 7B) during the ~4 min period (Figure 7C), were consistent with those reported in other studies (Han et al., 2020; Basnet et al., 2019; Beppi et al., 2021).

## 4 Discussion

Cisplatin has been used as an effective chemotherapy agent to treat a variety of solid tumors since the 1970s (Ghosh, 2019). However, despite its success, cisplatin is associated with adverse effects, including nephrotoxicity (which is reversible) and ototoxicity (which is irreversible), both of which compromise the quality of life for cancer survivors (Children’s Brain Tumor Foundation and Mattie Miracle Cancer Foundation, 2019; Fung et al., 2018; Karasawa and Steyger, 2015). Although there is a clear need for therapeutic interventions to prevent CIHL, it was only recently that STS was approved for use in a limited number of pediatric cancer patients (U.S. Food and Drug Administration, 2022).

Given cisplatin’s deleterious effects on the inner ear, many ototoxicity models have been developed with the goal of identifying therapies to prevent hearing loss (Karasawa and Steyger, 2015; Kros and Steyger, 2019). Two primary models have been frequently used in mice: an acute model (Ingersoll et al., 2020; Hazlitt et al., 2018), in which cisplatin is administered as a high single dose, and a chronic model (Fernandez et al., 2019; Breglio et al., 2017), in which cisplatin is given in low, multi-cycle doses. The acute model results in high mortality rates and significant variability in hearing threshold elevations, while the chronic model, although associated with minimal or no mortality, still exhibits variability in hearing thresholds, with some animals losing more than 20% of their body weight.

The major issue with all these models is the lack of consistency in the preparation of cisplatin solutions. This is crucial because researchers typically start with solid cisplatin, which needs to be dissolved into working solutions. Cisplatin is highly soluble in dimethylsulfoxide (DMSO), but DMSO also inactivates the drug (Hall et al., 2014; Raghavan et al., 2015). On the other hand, cisplatin has very low solubility in aqueous solutions (Kadokawa et al., 2021), requiring the preparation of low-concentration stock solutions and incubation at 37°C. This process introduces uncertainty about the amount of active cisplatin available and whether toxic metabolites have formed during incubation. Additional sources of variability include the source of the cisplatin (which affects its purity) and differences in the preparation process, which may vary between laboratories and individuals.

We addressed these challenges by optimizing Fernandez et al.’s multi-cycle protocol (Fernandez et al., 2019) and using clinical cisplatin to develop ototoxicity models in mice and zebrafish. The advantages of using clinical cisplatin instead of cisplatin powder are: (1) it is the same formulation used to treat cancer patients; (2) it is already dissolved in an aqueous solution (saline) and thus, ready-to use; and (3) it is of high quality and purity. Additionally, the clinical cisplatin solution is easy to obtain online with a prescription or through institutional pharmacies at a lower price compared to research-grade powdered cisplatin.

As expected, the new multi-cycle protocol using clinical cisplatin resulted in threshold elevations and hair cell and pre-synaptic ribbon loss, with a 3 mg/kg b.w. dose showing a similar effect to the 2.5 mg/kg b.w. dose used by Fernandez et al. (2019), suggesting that clinical cisplatin is more ototoxic. However, mice treated with clinical cisplatin did not experience significant body weight loss, indicating that the use of powder cisplatin may contribute to general toxicity due to impurities or inconsistencies (variation in concentration) in the preparation process.

Zebrafish exhibited a dose-dependent response to cisplatin, with an EC_50_ of ~350 μM after 6-h exposure, leading to a significant reduction in normal behavioral responses. Again, because clinical cisplatin was already dissolved in aqueous solution, there were no issues with the preparation or incorporation of clinical cisplatin into the fish water.

Similar to the existing mouse models, zebrafish models for cisplatin-induced ototoxicity have also been developed. Various concentrations, incubation times, and post-recovery periods have been tested, with some combinations proving more effective than others (Lee et al., 2022a; Vlasits et al., 2012; Mackenzie and Raible, 2012; Shin et al., 2013; Hong et al., 2013; Choi et al., 2013; Min et al., 2014; Niihori et al., 2015; Thomas et al., 2013; Vargo et al., 2017; Kitcher et al., 2019; Zheng et al., 2020). In most drug screening studies, cisplatin powder (e.g., Sigma, ThermoFisher) is used as the initial material, which is subsequently dissolved in DMSO or E3 media (Hong et al., 2013; Uribe et al., 2013; Lee et al., 2022b; Min et al., 2014; Niihori et al., 2015; Vlasits et al., 2012; Zheng et al., 2020). Both solvents can introduce undesirable effects: DMSO not only inactivates cisplatin (Hall et al., 2014), but also enhances its toxicity (Uribe et al., 2013), due to the formation of harmful metabolites. In contrast, published studies employing E3 media often fail to provide detailed information regarding cisplatin preparation (Hong et al., 2013; Choi et al., 2013; Min et al., 2014; Niihori et al., 2015). Given cisplatin’s poor solubility in aqueous solutions, additional steps, such as heating or sonication, are typically required to achieve a homogenous solution. Furthermore, a limited number of studies have used cisplatin from local pharmacies, but these sources lack specific details on cisplatin’s brand or grade (Thomas et al., 2013). These variations and omissions in the preparation procedures can introduce significant experimental variability. Furthermore, when considering differences in incubation times and cisplatin doses, substantial inter-laboratory variability is likely. Thus, the use of clinical cisplatin in the zebrafish ototoxicity model addresses these issues by reducing general toxicity (e.g., no DMSO) and minimizing variability between biological replicates (e.g., by ensuring more homogenous solutions). This leads to improved reproducibility and a reduction in the number of animals required to achieve statistical significance.

However, while the zebrafish lateral line model offers an accessible, rapid, and cost-effective alternative for studying ototoxicity, it is not without limitations. One key limitation is that, unlike mammalian hair cells, zebrafish hair cells possess regenerative capacity. Consequently, damage to zebrafish hair cells can trigger regeneration pathways, potentially complicating the interpretation of results in comparison to mammalian models. Thus, long-term incubation studies in zebrafish are difficult to perform due to mixed outcomes (regeneration *versus* protection). Another limitation is the absence of anatomical structures, such as the *stria vascularis* and the spiral ganglion neurons, which are likely affected by cisplatin-induced ototoxicity. Therefore, the zebrafish model limits conclusions to the hair cells and their surrounding supporting cells. Finally, while cisplatin affects high frequencies in the mammalian cochlea, zebrafish lack a frequency-based organization.

Nonetheless, given that the mechanisms of cisplatin-induced damage share similarities between zebrafish and mammalian hair cells, it represents a powerful model for screening of potential protective compounds, which can subsequently be tested in a mammalian models.

In summary, we have optimized two animal models for cisplatin-induced ototoxicity using clinical cisplatin. This approach offers a reliable methodology for identifying therapies to prevent hearing loss by minimizing cisplatin preparation variables and ensuring drug consistency and purity. This method could improve the translatability of preclinical findings to clinical settings, enhancing the development of protective treatments for cancer patients at risk of cisplatin-induced hearing loss.

## Data Availability

The original contributions presented in the study are included in the article/supplementary material, further inquiries can be directed to the corresponding authors.

## Glossary

ABRs: auditory brainstem responses
ANOVA: analysis of variance
BSA: bovine serum albumin
CDT: cumulative distance traveled
CIHL: cisplatin-induced hearing loss
CtBP2: C-terminal binding protein-2
DP: distortion product
dpf: days post-fertilization
DPOAEs: distortion product otoacoustic emissions
EC_50_: effective concentration causing 50% of the effect
FBS: fetal bovine serum
FDA: Food and Drug Administration
HCS1: hair cell soma-1
Hz: Hertz
IHC: inner hair cell
kHz: kiloHz
Lx: Lux
NIH: National Institutes of Health
OHC: outer hair cell
PBS: Phosphate Buffer Saline
PBSTx: PBS + Triton-X100
PFA: paraformaldehyde
ROS: reactive oxygen species
SD: standard deviation
STS: sodium thiosulfate
